# Supplementary material for: A high-resolution mRNA expression time course of embryonic development in zebrafish
Source: eLife. 2017 Nov 16;6:e30860. doi: 10.7554/eLife.30860 (PMC5690287; doi:10.7554/eLife.30860)
Supplement: Supplementary file 6. [file elife-30860-supp6.zip › biolayout-clusters-files/Cluster136-genes.html]

Cluster136


# Cluster136: Genes

| Ensembl ID | Gene Name | Chr | Start | End | Biotype |
| --- | --- | --- | --- | --- | --- |
| ENSDARG00000013500 | abca2 | 5 | 24583557 | 24732772 | protein\_coding |
| ENSDARG00000019613 | ip6k2b | 6 | 40092207 | 40165206 | protein\_coding |
| ENSDARG00000086985 | klc4 | 22 | 38498161 | 38530654 | protein\_coding |
| ENSDARG00000005481 | nfkbiaa | 20 | 16982300 | 16985154 | protein\_coding |
| ENSDARG00000101081 | ptprub | 16 | 34962068 | 35377055 | protein\_coding |
| ENSDARG00000016490 | rab11fip3 | 24 | 37835465 | 37905460 | protein\_coding |
| ENSDARG00000036773 | s100s | 19 | 7676901 | 7690361 | protein\_coding |
| ENSDARG00000016623 | si:ch211-195b13.1 | 19 | 25288110 | 25305061 | protein\_coding |
| ENSDARG00000075666 | tsc22d3 | 14 | 27700651 | 27703881 | protein\_coding |
